# Co-elevation of atmospheric [CO_2_] and temperature alters photosynthetic capacity and instantaneous water use efficiency in rice cultivars in a cold-temperate region

**DOI:** 10.3389/fpls.2022.1037720

**Published:** 2022-11-23

**Authors:** Chunyu Zhang, Yansheng Li, Zhenhua Yu, Guanghua Wang, Xiaobing Liu, Junjie Liu, Judong Liu, Xingmei Zhang, Kuide Yin, Jian Jin

**Affiliations:** ^1^ College of Agriculture, Heilongjiang Bayi Agricultural University, Daqing, China; ^2^ Key Laboratory of Mollisols Agroecology, Northeast Institute of Geography and Agroecology, Chinese Academy of Sciences, Harbin, China; ^3^ Centre for AgriBioscience, La Trobe University, Bundoora, VIC, Australia

**Keywords:** elevated [CO_2_], warming, *oryza sativa L.*, photosynthesis capacity, water use efficiency

## Abstract

Crop photosynthetic capacity in response to climate change likely constrains crop productivity and adaptability to changing environments, which requests the investigation on the dynamics of photosynthetic parameters over growth season among varieties, especially in cold-temperate regions. Three Japonica rice cultivars i.e., Shoubaimao (SH), Hejiang 19 (HJ); Longjing 31, (LJ). were planted under the control, e[CO_2_] (700 μmol mol^-1^), warming (2°C above the air temperature) and the co-elevation of [CO_2_] and temperature in open-top chambers (OTC). The objective of this study is to examine the rice photosynthetic parameters, water use efficiency (*WUE*) and yield formation in responses to the co-elevation of [CO_2_] and temperature which is the main predicted features of future climate. e[CO_2_] significantly increased *A_n_* of SH, HJ and LJ by 37%, 39% and 23% in comparison to 34%, 34% and 27% under elevated [CO_2_] plus warming, respectively. However, *A_n_* had a weak response to warming for three cultivars. [CO_2_] and temperature co-elevation significantly decreased the stomatal conductance, resulting in a significant increase of the *WUE*. e[CO_2_] significantly increased *V_c, max_*, *J_max_* and *J_max_*/*V_c, max_*. e[CO_2_] significantly increased grain yield and grain number of all cultivars. The positive effect of co-elevation of [CO_2_] and temperature on grain yield was less than e[CO_2_]. Warming is likely to partially offset the increased photosynthetic rate caused by e[CO_2_]. The [CO_2_] and temperature co-elevation may be favorable to rice crop with increasing the photosynthetic ability of rice crop and improving water use efficiency. The present study provided evidence that the rice genotypic difference in photosynthetic potential under [CO_2_] and temperature co-elevation. Therefore, it is crucial to explore a broader range of phenotypes and cultivars to be applied to climate change response research, advancing the knowledge that climate change impacts rice crop under the cold-temperate climate region.

## Introduction

The Earth System Model (ESM) in the IPCC’s Sixth Assessment Report (AR6) correctly predicted that the atmospheric CO_2_ concentration ([CO_2_]) would increase from 414 parts per million (ppm) in 2020 to over 1100 ppm by 2100 if no measures are implemented greenhouse gas emissions. Global warming is mostly attributed to the increase in atmospheric [CO_2_], which is expected to increase by 1.5 to 2°C in the end of the 21st century (IPCC, 2021). In the face of resource restrictions and the challenges of climate change, it is necessary to provide more food for the world’s rapidly growing population, whereas crop production has been increasing at a decreasing rate, which has largely motivated contemporary photosynthesis research.

Increased [CO_2_] levels and warming are experiencing a rapid detrimental effect on ecosystems around the world, particularly in agricultural ecosystems, causing changes in plant growth and nutrient acquisition (Carvalho et al., 2020; Guo et al., 2022). As the fundamental substrate in the process of photosynthesis, substantial pieces of evidence have indicated that e[CO_2_] will stimulate photosynthesis and benefit biomass production and yield, particularly in C_3_ crops (Cheng et al., 2009; Zhu et al., 2016; Lenka et al., 2019; Brito et al., 2020; Lenka et al., 2021b; Zhu et al., 2022). Considering the warming effect, there are inconsistent conclusions on crop biomass and yield. Previous works have indicated that warming suppressed photosynthesis and shortened the plant growth period, resulting in a reduction of crop biomass (Bhattacharyya et al., 2014; Wang et al., 2016; Cai et al., 2016). However, contrary results have also been reported that warming increased plant growth due to the stimulation of metabolic activities (Arcus et al., 2016; Dusenge et al., 2019). In other words, the optimal temperature for the growth of rice, as a typical thermophilic C_3_ crop, can reach 35°C, and plants even tolerate 40°C in the vegetative growth stages (Bahuguna et al., 2015; Wang et al., 2015b). Varietal variation and climate region should also be considered to reveal the crop response to climate change.

The productivity of crops depends on physiological and biochemical capacity of photosynthesis. The Farquhar - von Caemmerer - Berry model (the FvCB model, Farquhar et al., 1980) is a method applied to studies on climate-plant, that of two key parameters: the maximum rates of electron transport (*J_max_*) and carboxylation by Rubisco (*V_c, max_*) are obtained through the measurement and modeling of photosynthetic response to CO_2_ concentration (*A_n_*-*C_i_* curves) (Rogers, 2014; Coursolle et al., 2019; Miao et al., 2021). As far as we know, only one report by Cai et al. (2018) was carried out in the subtropical region, and showed that the effect of e[CO_2_] and warming on *A_n_*-*C_i_* curves were variable among different growth stages of rice. The results demonstrated that *V_c, max_* and *J_max_* significantly decreased under the simultaneous increase of CO_2_ concentration and temperature at the heading and filling stages. Ecosystem modeling may contribute to the understanding of biochemical responses to the interaction between the plant and the future environment (Le et al., 2011; Drewry et al., 2014).

Rice (*Oryza sativa* L.) is one of the most important sources of daily food for nearly half of the world’s population (Zhu et al., 2018). It is imperative to examine and predict the responsive changes in the physiology and growth of rice to CO_2_ enrichment and warming. Rice is grown in paddy soils, which maintain a relatively consistent water depth throughout the growing season (Thanawong et al., 2014; Bocchiola, 2015). Water use is continuously concerned under climate change because it reflects the coupled relationship between water loss and carbon gain in the process of plant photosynthetic carbon assimilation (Ullah et al., 2019). Clear pieces of evidence indicated that e[CO_2_] directly decreased leaf stomatal conductance by approximately 50%, reducing water losses and increasing water use efficiency (Yoshimoto et al., 2005; Ainsworth and Rogers, 2007). Qiao et al. (2010) found that the water use efficiency of C_3_ crops was promoted by e[CO_2_] but not by warming. However, the reduction in stomatal conductance in response to e[CO_2_] can vary depending on plant status, such as growth stage, as well as environmental factors, such as light and temperature (Bunce, 2004). Some recent investigations have concluded that stomatal conductance responds to e[CO_2_] and warming without a consistent trend (Del Pozo et al., 2005; Mott and Peak, 2013; Urban et al., 2017). Therefore, to date, very limited information is available on the impact of [CO_2_] and warming co-elevation on stomatal conductance of rice, which would provide new insights into potential agricultural manipulations to reduce water use under future climatic conditions.

The local climate of Northeast China is characterized as a cold temperate and humid and sub-humid climate, where the rice area is expanding rapidly with an increasing rate of 0.16 ×10^6^ ha per year (Xin et al., 2020). Crop productivity is being impacted by climate change. However, as the result of climate change variables, most research focused on photosynthesis responses in the grain yield processes to e[CO_2_] or elevated temperature instead of their interactions. Previous studies have been carried out in tropical regions, demonstrating that warming negatively affected rice yield (Wang et al., 2016; Wang et al., 2020a; Wang et al., 2020b). We hypothesize that (1) in cold-temperate climate regions, elevated air temperatures within a 2°C increment may not negatively impact rice growth and physiological traits because the temperature is below the critical level during rice growing season; (2) [CO_2_] and temperature co-elevation would improve photosynthesis with an increase in water use efficiency, thereby contributing to rice production. In this study, the photosynthesis curve response to [CO_2_] and its parameters were measured in three rice cultivars grown in the open-top chambers. Seasonal variations of transpiration rate and water use efficiency were examined at different growing stages under e[CO_2_] and increased temperature. The primary objectives of this study were to (1) investigate seasonal and varietal variations of rice leaf photosynthetic performance under [CO_2_] and temperature co-elevation and (2) examine whether elevated temperature could benefit yield formation and increase rice productivity when combined with e[CO_2_] in a cold-temperate region. The present study’s findings will be conducive to further understanding the response of yield to e[CO_2_] and temperature and their interaction in a cold-temperate region of China, and can support decision making for adaptation strategies in rice production under climate change.

## Materials and methods

### Experimental design

This experiment was conducted in open-top chambers (OTCs) at the Northeast Institute of Geography and Agroecology, Chinese Academy of Sciences, Harbin, China (45°73′N, 126°61′E). A pot experiment was carried out with three rice cultivars and four different environmental treatments. The treatments comprised ambient [CO_2_] (400 μmol mol^-1^) and temperature (Control), elevated [CO_2_] (700 μmol mol^-1^, e[CO_2_]), warming (+2°C), and e[CO_2_] plus warming (e[CO_2_]+warming). The ambient temperature during the growth season was in a range of 14.2–27.9°C. The structure of OTC was 3.5 m in diameter and 2.0 m in height, with a 0.5 m high canopy that made a 45° angle to the horizontal plane. Covering in OTCs was done with a clear polyethylene film. Zhang et al. (2014) and Li et al. (2018); Li et al. (2019b) illustrated the OTC design in further details. The CO_2_ concentration inside OTCs was monitored and automatically controlled by supply of 99.9% CO_2_ gas using a computerized CO_2_-regulating system (Beijing VK2010, China). The inner temperature in each OTC was effectively regulated *via* an air conditioner with a pair of thermal probes installed inside and outside of the OTC in order to maintain the temperature inside 2°C higher than or as same as outside. In addition, an inner air flow system was installed to homogenize the CO_2_ concentration and temperature in the OTCs.

### Plant material and growth conditions

Three rice cultivars cv. Shishoubaimao (SH), Hejiang 19 (HJ), and Longjing 31 (LJ) were bred over the historical period, i.e. approximately 7 decades and have been grown on a large scale in the cold-temperate climate region (**Table 1**). As the first generation, SH was introduced from Japan to northeast China in the 1940s, and then the cultivars HJ and LJ were subsequently bred (**Table 1**). Rice plants were grown in pots and randomly allocated in OTCs, with three pots for each cultivar under each treatment. The paddy soil is classified as a typical Mollisol (USDA). The soil was collected from field sites in Hailun City, Heilongjiang, China (47°23′N, 126°51′E). The air-dried soil was sieved through a 4-mm sieve and mixed well. Nine kg of sieved soils were placed into each pot (height 23 cm, diameter 30 cm). Basal nutrients (mg nutrient per kilogram soil) of 90 mg kg^-1^ of N, 113 mg kg^-1^ of P_2_O_5_, and 74 mg kg^-1^ of K_2_O were applied before transplanting. Uniform rice seeds were germinated and grown in a tray filled with 5 cm of soil in depth. On the 20th day after emergence, three seedlings of rice with similar size were transplanted into each pot. The paddy soil was always submerged under 3-4 cm of water. The elevated CO_2_ concentration and temperature treatments were sustained until the end of the experiment.

**Table 1:** Cultivar name, year of release or introduction, sub-species and days from transplanting to maturity for the chosen three rice cultivars.

Cultivar name	Breeding locations	Year of release or introduction	Sub-species	Days from transplanting to maturity
Shishoubaimao	Hokkaido, Japan	1935	*subsp.japonica*	125
Hejiang 19	Heilongjiang, China	1978	*subsp.japonica*	125-130
Longjing 31	Heilongjiang, China	2011	*subsp.japonica*	130

### Leaf photosynthetic parameter measurements

As reported in the previous studies, the tiller stage is a critical stage when the biomass rapidly accumulated and panicles form. The anthesis stage is more sensitive to environmental factors, such as temperature, light and soil water content. Similarly, carbohydrates are transferred from the source (shoot) to the pool (seed) during the grain filling stage (Jagadish et al, 2007; Sasaki et al, 2007; Wassmann et al., 2009). The varietal response to the climate change would be maximal over those growth stages. Therefore, measurements and samples were taken at the three stages of those cultivars. The three youngest fully expanded leaves (flag leaves at the anthesis and grain-filling stage) were tagged in each treatment for measurement. Photosynthetic measurements were performed during the period from 8:30 to 11:30. We used the LI-Cor 6800 Portable Photosynthesis System (Li-Cor BioScience, Lincoln, NE, USA) with Dynamic Assimilation™, and 6800-01A leaf chamber fluorometers to measure the net photosynthetic rate (*A_n_*) in response to intercellular carbon dioxide concentration (*C_i_*) by controlling the CO_2_ concentration in the leaf chamber (*A_n_*-*C_i_* curves). Detailed information about instructions for equipment and programs was described by Saathoff and Welles (2021). Measurement temperature was set as same as the ambient temperature for the control and e[CO_2_] treatments, and +2°C above ambient temperature for warming and e[CO_2_] plus warming (e[CO_2_]+warming) treatments. Other leaf chamber conditions were as follows: saturating irradiance of 1200 μmol m^-2^ s^-1^, the fan speed of 10 000 rpm, the flow rate of 500 μmol mol^-1^, and relative humidity at leaf of 60%. Steady-state values of *A_n_* (μmol CO_2_ m^-2^ s^-1^), *G_s_* (mol H_2_O m^-2^ s^-1^), intercellular [CO_2_] (*C_i_*, μmol mol^-1^), ambient [CO_2_] (*C_a_*, μmol mol^-1^) were recorded during each measurement.

The *A_n_*-*C_i_* curves. The LI-6800’s Auto Program was used to process: a “down” ramp from 400 to 50 μmol mol^-1^ at a rate of 400 μmol mol^-1^ min^-1^ CO_2_ and was immediately followed, approximately 10 to 15 s later, by an “up” ramp from 50 to 1 600 μmol mol^-1^at a rate of 400 μmol mol^-1^min^-1^. The data was recorded on a computer every 2 s in all measurements. In the measurement of *A_n_*-*C_i_* curves, stomatal conductance (*G_s_*), transpiration rate (*T_r_*), and air -to- leaf temperature difference (*ΔT*) were also automatically measured. The instantaneous water use efficiency (*iWUE*) is the ratio of *A_n_* and *T_r_*.

### Leaf chlorophyll content measurements

In order to extract chlorophyll, 0.3 g of fresh leaf material was stored in 15 ml of 80% (v/v) ethanol for more than 2 days in the dark at 4°C. The absorbance of the solution was measured using a multi-functional enzyme labeler (CLARIOstar Plus, Germany) at 645 and 663 nm. The *Chl a* and *Chl b* content were calculated according to the equations of Arnon (1949) using the absorbance values.

### Plant harvest and measurements

The plants were destructively sampled at the tillering, anthesis and grain filling stages. Leaves, stems, and panicles (when present) were separated from samples. After that, the samples were dried at 105°C for 30 min and then dried at 75°C for 72 h, after which the biomass components were recorded. At maturity, grain was harvested to measure grain yield and its components.

### Data processing and statistical analysis

The *A_n_*-*C_i_* curves were averaged across three repetitions for the given experiment treatment before further analyses. Using the ‘plantecophys’ package (Duursma, 2015) in R version 3.6.1 (R Core Team, 2019), *A_n_*-*C_i_* curves were fit to the FvCB model to estimate the photosynthetic parameters including CO_2_ compensation point (*Γ*, µmol mol^-1^), maximum ribulose -1, 5-bisphosphate carboxylase/oxygenase (Rubisco) carboxylation rate (*V_c, max_*, µmol m^-2^ s^-1^), and potential light saturated electron transport rate (*J_max_*, µmol m^-2^ s^-1^), respectively. All settings in the ‘fitaci’ function were left at their respective default values, and the default nonlinear fitting method was used, which allows for the calculation of standard errors.

A one-way ANOVA (analysis of variance) was conducted to determine the effects of e[CO_2_], warming and their interactions on *Γ*, *V_c,max_*, and *J_max_*. Treatment means were compared using the Duncan’s multiple range test (*p* < 0.05) in SPSS 22.

## Results

### The phenotypes and gas exchange under e[CO_2_] and warming

**Figure 1:**
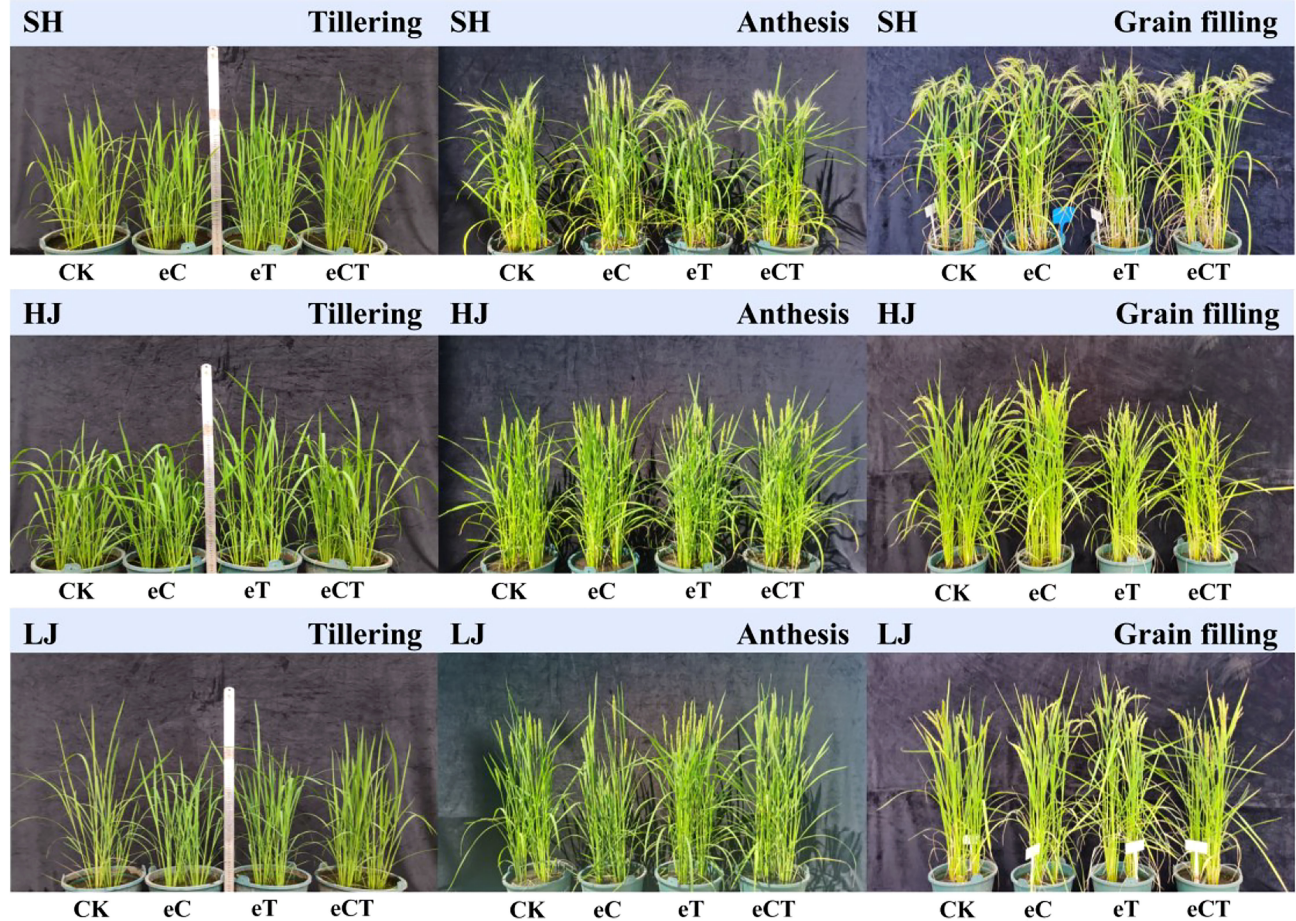
The rice growing conditions under different treatments during the stages of tillering, anthesis and grain filling (control, ambient conditions; eC, eCO_2_ concentration; eT, elevated air temperature; eCT, the combination of elevated air temperature and [CO_2_]) at three growth stages (tillering, anthesis and grain filling). SH, HJ and LJ refer to three rice cultivars Shishoubaimao, Hejiang 19, and Longjing 31, respectively.

Warming increased plant height. The treatments had no significant effect on plant height at the anthesis and grain filling stages, except for HJ at the grain filling stage that warming alone and e[CO_2_] plus warming decreased plant height, compare to control (**Figure 1**).

**Figure 2:**
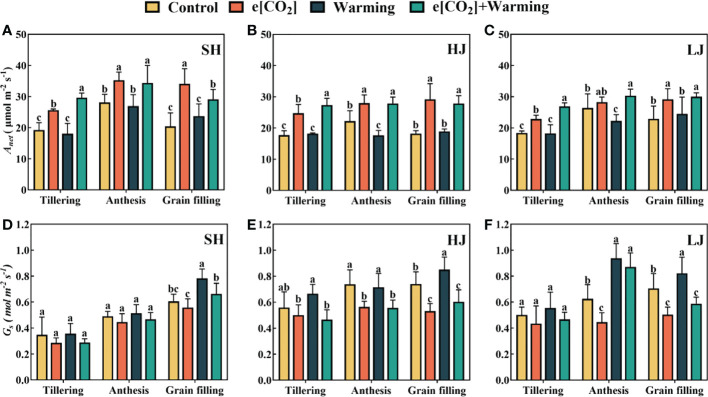
The responses of *A_n_* **(A–C)** and *G_s_* **(D–F)** measured under control, ambient condition; e[CO_2_], eCO_2_ concentration; warming, elevated air temperature; e[CO_2_] plus warming, the combination of elevated air temperature and [CO_2_] during the stages of tillering, anthesis and grain filling. SH, HJ and LJ refer to three rice cultivars Shishoubaimao, Hejiang 19, and Longjing 31, respectively. Values are the means ± SEs (n = 6). Different lower-case letters indicate significant differences between treatments at *P* < 0.05 level. *A_n_*, net photosynthetic rate; *G_s_*, stomatal conductance.

Compared to the control, e[CO_2_] resulted in significant increases in leaf *A_n_* at three sampling stages, in SH, HJ and LJ, ranging from18%-67%, 26%-60% and 11%-37%, respectively (**Figure 2**). Compared to the control, HJ and LJ leaf *A_n_* at the anthesis significantly decreased under warming by 20.6% and 15.6% respectively. Compared to the control, e[CO_2_] plus warming increased *A_n_* of SH, HJ and LJ by 15%-53%, 18%-47% and 11%-37% throughout all three stages. Compared to control, warming resulted in an increase in leaf *G_s_* among all cultivars. It is interesting to note that e[CO_2_] alleviated this phenomenon. e[CO_2_] and e[CO_2_] plus warming decreased leaf *G_s_* (**Figure 2**).

**Figure 3:**
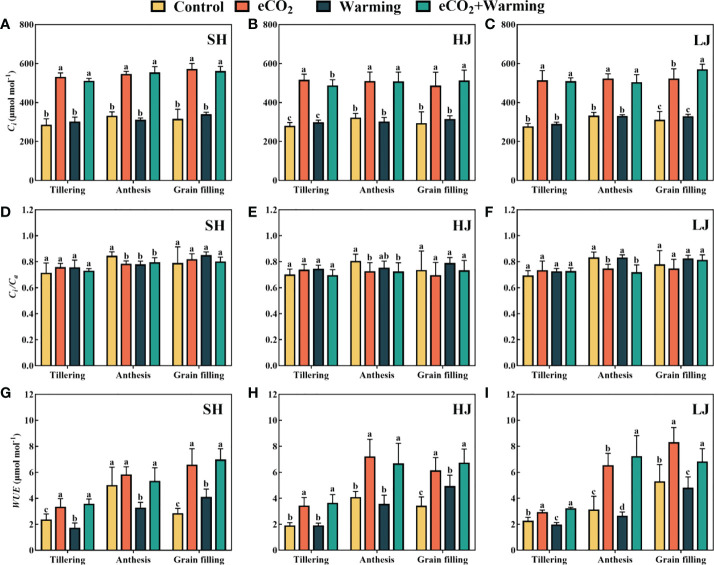
The value of *C_i_* **(A-C)**, *C_i_*/*C_a_* **(D-F)** and *WUE* **(G-I)** of three rice cultivars grown under control, ambient conditions; e[CO_2_], elevated CO_2_ concentration; warming, elevated air temperature; e[CO_2_] plus warming, the combination of elevated air temperature and [CO_2_]). SH, HJ and LJ represent rice cultivars Shishoubaimao, Hejiang 19, and Longjing 31, respectively. Values are the means ± SEs (n = 6). Different letters indicate significant differences between treatments at *P* < 0.05 level. *C_i_*, intercellular [CO_2_]; *C_i_*/*C_a_*, the ratio of intercellular [CO_2_] to ambient air [CO_2_]; *WUE*, water use efficiency.

Compared to the control, warming had no significant effect on *C_i_*, but it increased significantly under e[CO_2_] and e[CO_2_] plus warming among all cultivars (**Figure 3**). e[CO_2_] and e[CO_2_] plus warming significantly decreased *C_i_*/*C_a_* at the anthesis stage among all cultivars. e[CO_2_] plus warming had a positive effect on the *WUE*, and warming weakened the stimulation of *WUE* caused by e[CO_2_] was observed at the anthesis stage of SH and grain filling stage of HJ.

### CO_2_ response curves and model parameters

**Figure 4:**
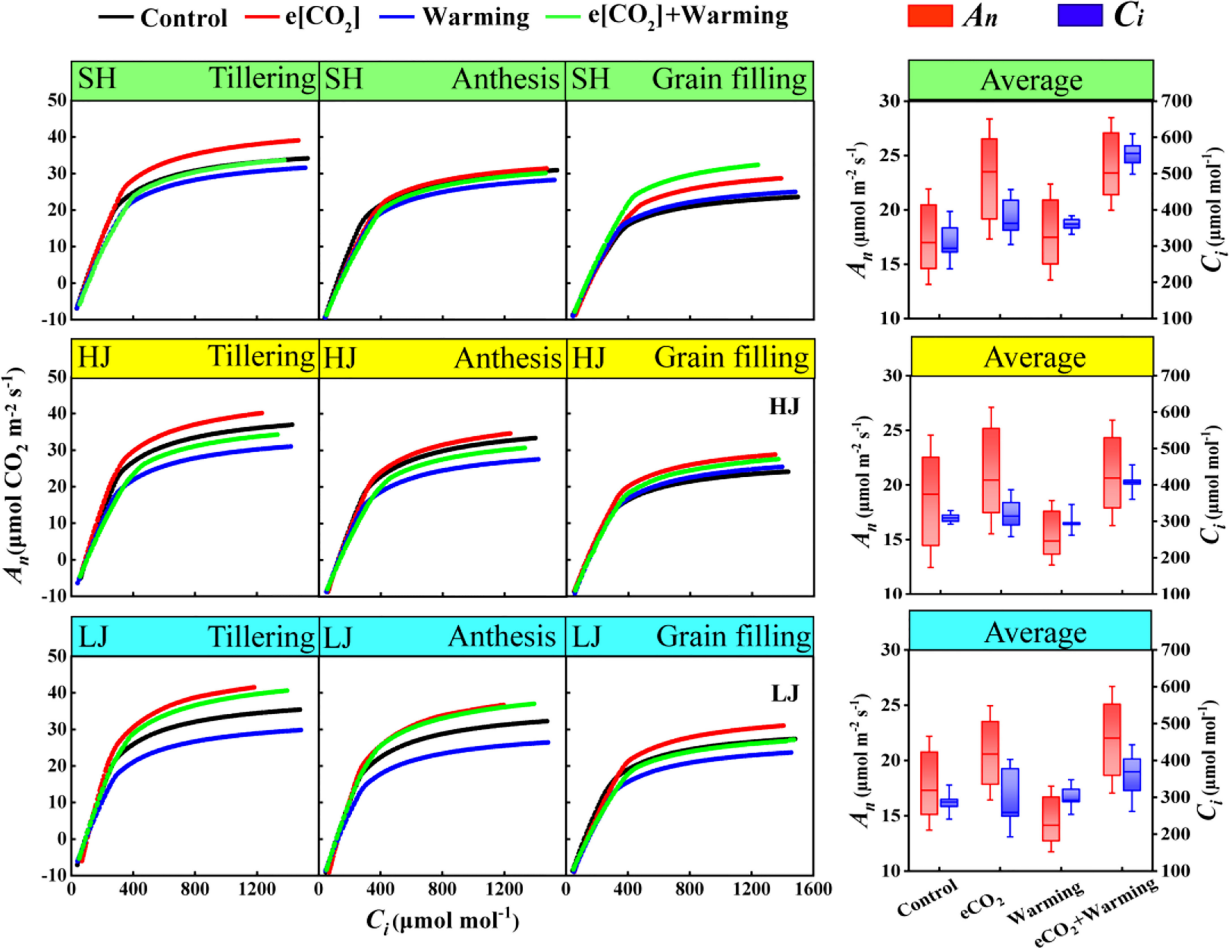
The line graph represents the net photosynthetic rate (*A_n_*)- intercellular carbon dioxide concentration (*C_i_*) curves under ambient [CO_2_] and temperature (black dots, Control), e[CO_2_] (red dots, e[CO_2_]), warming (blue dots, warming), and e[CO_2_] plus warming (green dot, e[CO_2_] +warming) at the tillering, anthesis, and grain-filling stages (from left to right) in SH (Shishoubaimao), HJ (Hejiang 19), and LJ (Longjing 31) (from top to bottom). The box graph represents the average net photosynthetic rate (*A_n_*, red boxes) and intercellular carbon dioxide concentration (*C_i_*, blue boxes) when the carboxylation rate is maximum.

**Figure 5:**
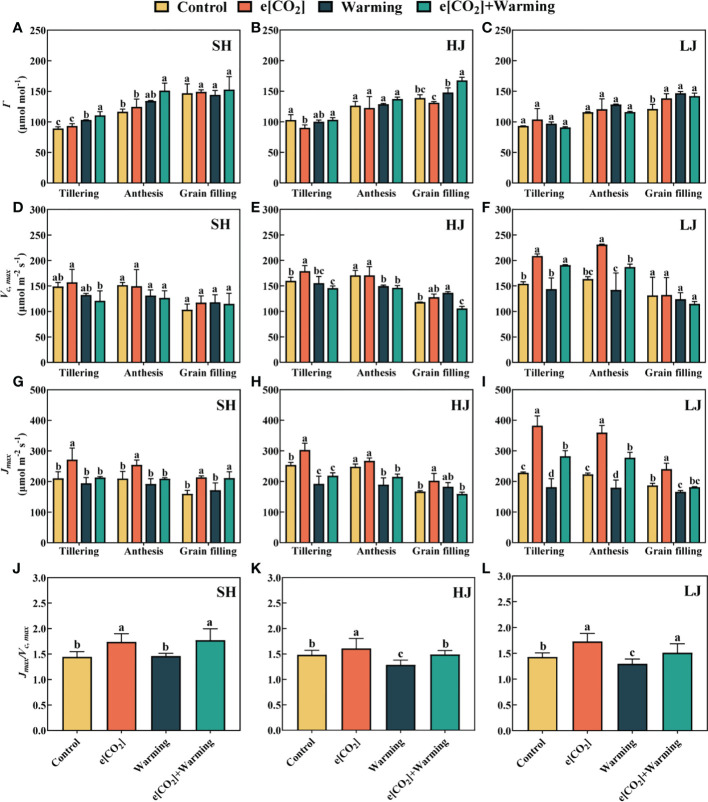
Model parameters were determined from *A_n_*-*C_i_* data. Carbon dioxide compensation point (*Γ*, **A–C**), maximum ribulose-1, 5-bisphosphate carboxylase/oxygenase (Rubisco) carboxylation rate (*V_c, max_*, **D–F**), and potential light saturated electron transport rate (*J_max_*, **G–I**) under e[CO_2_] and warming at the tillering, anthesis, and grain-filling stages of SH (Shishoubaimao), HJ (Hejiang 19), and LJ (Longjing 31). Data are means ± standard errors (n = 3). The data of (*J_max_*/*V_c, max_*, **J–L**) radio is the average means ± standard errors (n = 9) at three stages in each cultivar. Different letters indicate significant differences between mean values at *P* < 0.05 level.

Compared with the control, e[CO_2_] increased *A_n_* across three varieties at three stages, after the carboxylation rate reaches its maximum (**Figure 4**). However, warming decreased *A_n_* across three varieties, but not for SH and HJ at the grain-filling stage. The stimulating effect of e[CO_2_] on *A_n_* was counteracted by warming.

e[CO_2_] plus warming increased *C_i_* and *A_n_* by 43% and 38% at the maximum of the carboxylation rate throughout three stages of SH, respectively, compared with the control (**Figure 4**). A similar trend was found with a 31% and 14% of increase for *C_i_* of HJ and maximal *A_n_*, and 18% and 22% of LJ, respectively (**Figure 4**).

e[CO_2_] plus warming significantly (*P* < 0.05) increased *Γ* by 24% and 30% at the tillering and anthesis stages of SH (**Figure 5**), by 21% and 17% at the grain-filling stage of HJ and LJ compared with the control, respectively. The effect of e[CO_2_[ plus warming on *Γ* varied among the three varieties and stages.

The response of maximum ribulose -1, 5-bisphosphate carboxylase/oxygenase (Rubisco) carboxylation rate (*V_c, max_*) to climate change depended on varieties and growth stages (**Figure 5**). e[CO_2_] plus warming significantly decreased *V_c, max_* by 8.9% and 14% at the tillering and anthesis stages of HJ, while significantly increased *V_c, max_* by 24% at the grain-filling stage of LJ. e[CO_2_] also increased the *V_c, max_* at the tillering and anthesis stages of LJ.

Similarly, *J_max_* increased by 32% in response to e[CO_2_] plus warming at the grain-filling stage of SH (**Figure 5**), and increased by 24% and 24% of LJ but reduced by 13% and 14% at the tillering and anthesis stages of HJ, respectively, compared with control. Compared with the control, e[CO_2_] significantly increased the average *J_max_*: *V_c, max_* ratio across three varieties at three stages.

### Leaf chlorophyll content

**Figure 6:**
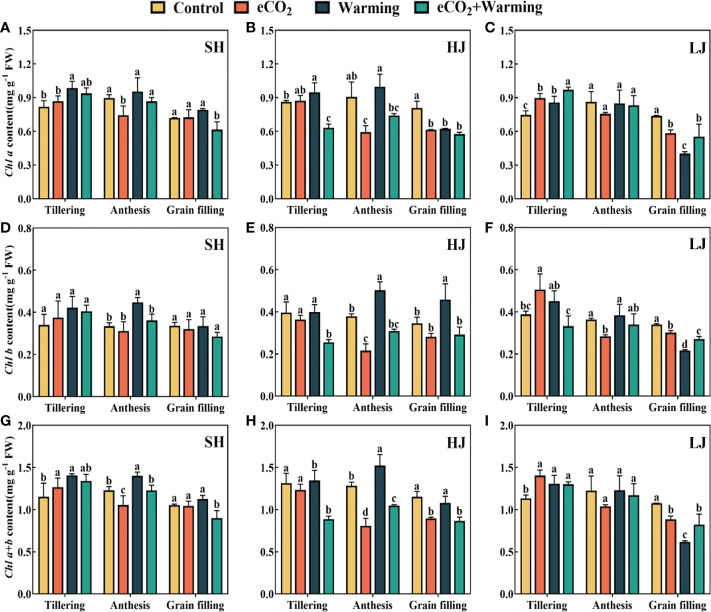
The leaf chlorophyll a (*Chl a*, **A-C**), chlorophyll b (*Chl b*, **D-F**) and chlorophyll a+b (*Chl a+b*, **G-I**) under control, ambient conditions; e[CO_2_], elevated [CO_2_] concentration; warming, elevated air temperature; e[CO_2_] plus warming, the combination of elevated air temperature and [CO_2_]) at the growth stage of tillering, anthesis and grain filling. SH, HJ and LJ refer to three rice cultivars Shishoubaimao, Hejiang 19, and Longjing 31, respectively. Values are the means ± SEs (n =6 ).Different lower-case letters indicate significant differences between treatments at *P* < 0.05 level.

Compared to the control, the leaf chlorophyll a (*Chl a*) content under e[CO_2_] and warming in three cultivars had an average increase of 10% and 15%, respectively (**Figure 6**). Whereas the effect of e[CO_2_] plus warming on the leaf *Chl a* content varied among the varieties. For example, e[CO_2_] plus warming significantly decreased the leaf *Chl a* content by 18%-25% at the grain filling across the three varieties. The effect of climatic treatments on chlorophyll b (*Chl b*) and chlorophyll a+b (*Chl a+b*) is similar to that of *Chl a.*

### Dynamic response of stomatal conductance to *C_i_*

**Figure 7:**
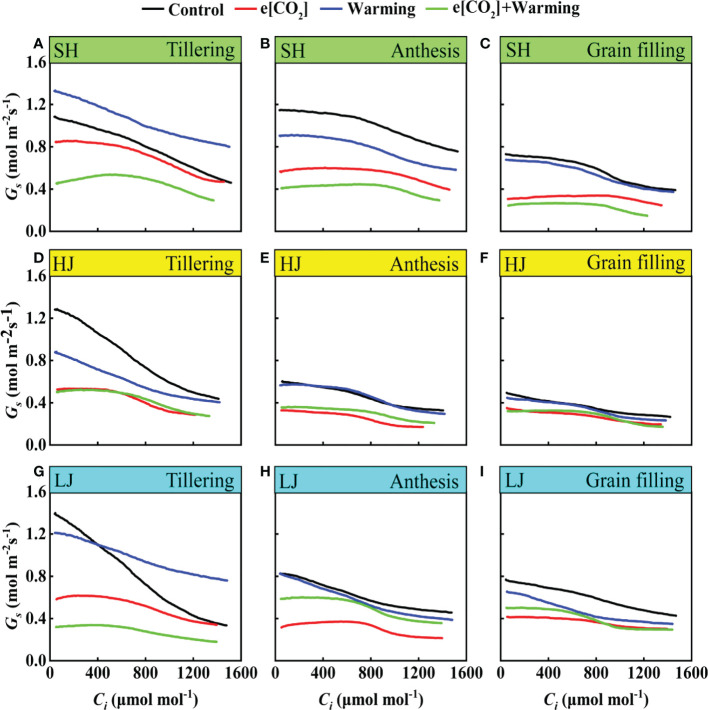
The *G_s_*-*C_i_* Curves under ambient CO_2_ and temperature (black dots, Control), elevated CO_2_ concentration (red dots, e[CO_2_]), elevated temperature (blue dots, warming), and elevated CO_2_ concentration plus warming (green dot, e[CO_2_] + warming) for cv. SH (**A-C**), HJ (**D-F**), and LJ (G, H, I) during the tillering stage, anthesis stage, and grain-filling stage. *G_s_* represents stomatal conductance, *C_i_* represents intercellular carbon dioxide concentration.

The *G_s_* tended to decrease with the increase of *C_i_*. Compared with the control, e[CO_2_] plus warming notably decreased *G_s_* irrespective of rice variety and growth stage (**Figure 7**). Interestingly, *G_s_* gradually decreased over the growing season, meanwhile, the effect of e[CO_2_] plus warming on *G_s_* reduced over time.

### Transpiration rate changes in response to e[CO_2_] and warming

**Figure 8:**
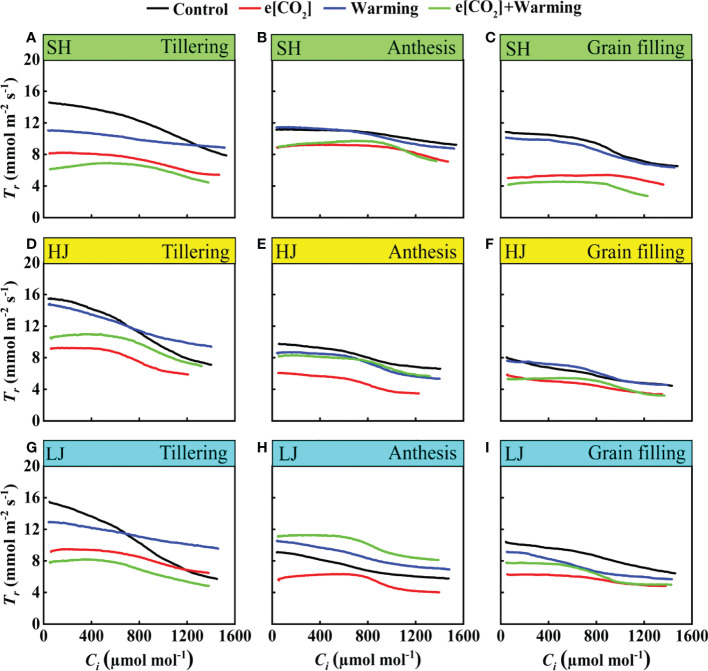
The *T_r_*-*C_i_* curves under ambient CO_2_ and temperature (black dots, Control), elevated CO_2_ (red dots, e[CO_2_]), elevated temperature (blue dots, warming), and eCO_2_ plus warming (green dot, e[CO_2_]+warming) for cv. SH (**A-C**), HJ (**D-F**), and LJ (**G-I**) during the tillering stage, anthesis stage, and grain-filling stage. *T_r_* represents transpiration rate, *C_i_* represents intercellular carbon dioxide concentration.

With the advancement of *C_i_*, the values of transpiration rate (*T_r_*) gradually decreased across three varieties. Except for the anthesis stage, e[CO_2_] plus warming significantly decreased *T_r_* (**Figure 8**) across three growth stages, compared with the control. Interestingly, with the growth period, the response of *T_r_* to climate change gradually weakened.

### Response of instantaneous water use efficiency under e[CO_2_] and warming

**Figure 9:**
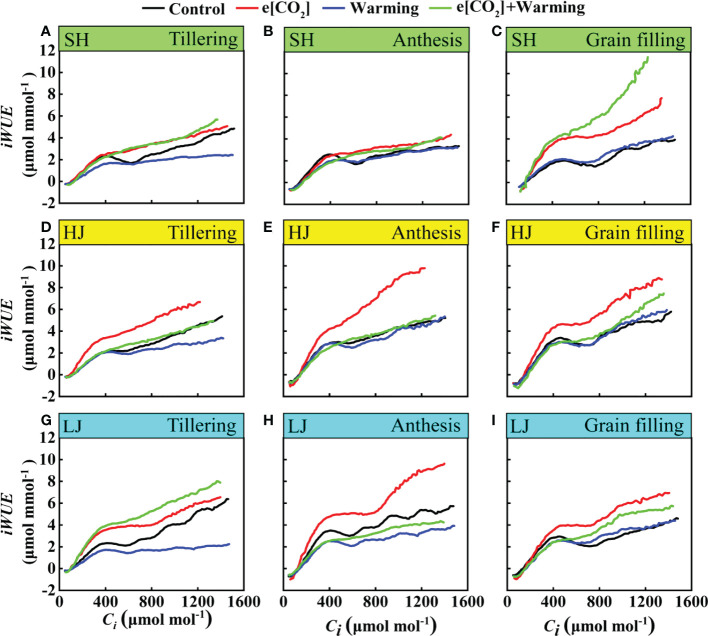
The *iWUE*-*C_i_* Curves under ambient [CO_2_] and temperature (black dots, Control), elevated [CO_2_] (red dots, e[CO_2_]), elevated temperature (blue dots, warming), and e[CO_2_] plus warming (green dot, e[CO_2_]+warming) for cv. SH (**A-C**), HJ (**D-F**), and LJ (**G-I**) during the stage of tillering anthesis, and grain-filling. *iWUE* represents instantaneous water use efficiency, *C_i_* represents intercellular carbon dioxide concentration.

In the present study, instantaneous water use efficiency (*iWUE*) in response to climate change varied among three varieties (**Figure 9**). With an increase in the intercellular carbon dioxide (*C_i_*), instantaneous water use efficiency of rice leaves showed an increasing trend with a greater increase under e[CO_2_] plus warming than the control, except for the anthesis stage of LJ. Taken together, these results indicated that climate change could pose a positive effect on the *iWUE*.

### Temperature differences between air and leaves in response to e[CO_2_] and warming

**Figure 10:**
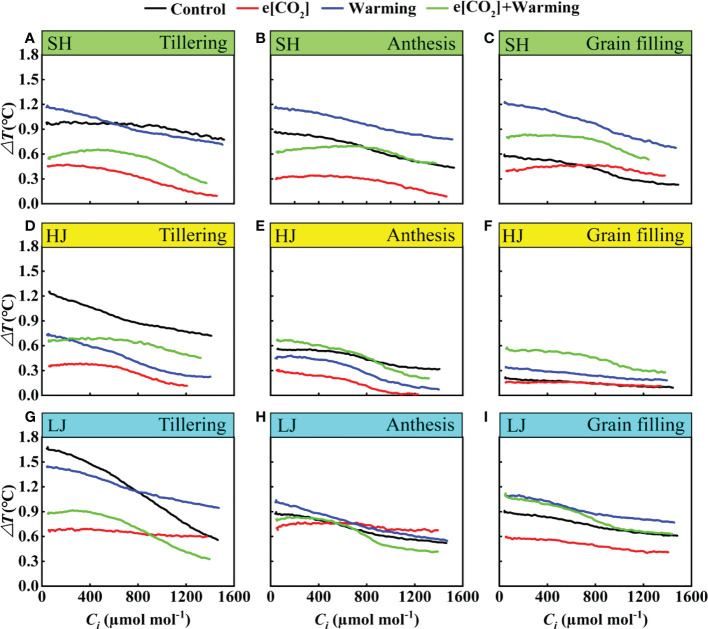
The *ΔT*-*C_i_* Curves under ambient CO_2_ and temperature (black dots, Control), elevated [CO_2_] (red dots, e[CO_2_]), elevated temperature (blue dots, warming), and e[CO_2_] plus warming (green dot, e[CO_2_] +warming) for cv. SH (**A-C**), HJ (**D-F**), and LJ (**G-I**) during the tillering, anthesis, and grain-filling stages. *ΔT* represents the air-to-leaf temperature difference, *C_i_* represents intercellular carbon dioxide concentration.

The air-to-leaf temperature difference of all treatments showed a gradual decrease with the increase of *C_i_* (**Figure 10**). Here, a clear reduction of *ΔT* was observed at the tillering stage across three varieties under e[CO_2_] plus warming, but there is little difference at the anthesis stage. However, compared to the control, e[CO_2_] plus warming notably increased *ΔT* across three varieties at the grain-filling stage. e[CO_2_] plus warming altered the trend that air-to-leaf temperature difference decreased gradually with the reproductive process.

### Aboveground biomass, grain yield and its components

**Figure 11:**
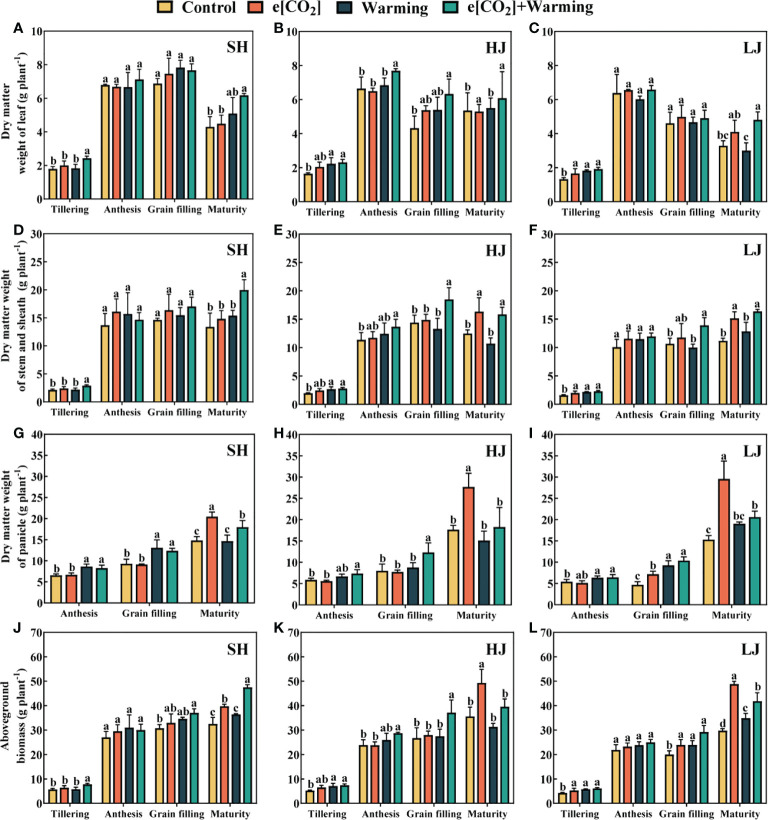
The dry matter weight of leaf **(A–C)**, stem **(D–F)**, panicle **(G–I)** and aboveground plant **(J–L)** at the stage of tillering, anthesis, grain filling and maturity under control, ambient conditions; e[CO_2_], elevated [CO_2_]; warming, elevated air temperature; e[CO_2_] plus warming, the combination of elevated air temperature and [CO_2_]. Values are the means ± SEs (n=3). Lower-case letters indicate significant differences between treatments at *P* < 0.05 level.

In comparison to the control, the aboveground biomass at the mature stage tended to increase under e[CO_2_], warming, and e[CO_2_] plus warming. (**Figure 11**). In general, the aboveground biomass of SH, HJ, and LJ increased by 28%, 28%, and 36% under e[CO_2_] plus warming. At maturity, the dry matter weight of leaves, stems, and panicles under e[CO_2_] plus warming increased by 13%-50%, 21%-73%, and 4%-34%, respectively, compared to the control.

In most cases, leaf dry weight decreased slightly with warming, which could offset the positive effect of e[CO_2_] on rice dry matter accumulation. For example, compared to e[CO_2_], a 12%-34% reduction of the panicle dry matter weight was observed under e[CO_2_] plus warming.

**Figure 12:**
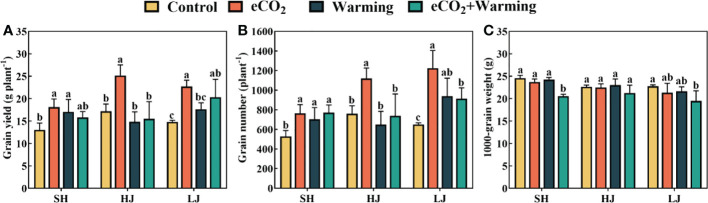
The rice yield **(A)**, grain number **(B)** and 1000-grain weight **(C)** of three rice cultivars grown under control, ambient conditions; e[CO_2_], elevated CO_2_ concentration; warming, elevated air temperature; e[CO_2_] plus warming, the combination of elevated air temperature and [CO_2_]. SH, HJ and LJ refer to three rice cultivars Shishoubaimao, Hejiang 19, and Longjing 31, respectively. Values are the means ± SEs (n = 3). Lower-case letters indicate significant differences between treatments at *P* < 0.05 level.

In comparison to the control, e[CO_2_] significantly increased grain yields of SH, HJ and LJ by 39%, 46% and 59%, respectively (**Figure 12**). Rice yield did not change significantly in SH and LJ under warming, but increased by 11% in HJ, compared to the control. The increase in rice yield under e[CO_2_] plus warming was less than under e[CO_2_] but higher than that under warming. Furthermore, e[CO_2_] resulted in increases of 44%, 47%, and 88% in grain number in SH, HJ, and LJ. In contrast to the control, 1000-grain weight did not significantly differ among treatments except for a decrease under e[CO_2_] plus warming.

## Discussion

### *V_c, max_* and *J_max_* in response to e[CO_2_] and temperature

Previous studies demonstrated that e[CO_2_] led to a decrease in *V_c, max_* (Medlyn et al., 1999; Ainsworth and Long, 2005; Cai et al., 2018). For example, a FACE study by Cai et al. (2018) indicated a significant reduction in *V_c, max_* at several growth stages due to the interaction effect of e[CO_2_] (104-199 μmol/mol) and increased canopy temperature (1.0-1.7°C). Moreover, abiotic stress may constrain the effect of e[CO_2_] on *V_c, max_* and *J_max_*, as Liu et al. (2018) found that 7 days of e[CO_2_] increased cucumber *V_c, max_* and *J_max_* by 12.0% and 14.7%, respectively, but this effect disappeared under mild and severe drought stresses (Liu et al., 2018). However, in this study the *V_c, max_* and *J_max_* increased in response to e[CO_2_] and/or warming treatments, indicating that e[CO_2_] and warming favor rice photosynthetic potential in cold temperate regions. It is probably because the increased temperature in the warming treatment is still in the range of temperature for optimal growth of rice (Zhang et al., 2022).

The *V_c, max_* and *J_max_* in FvCB in response to [CO_2_] and temperature co-elevation varied among rice varieties. In this study, [CO_2_] and temperature co-elevation significantly increased *V_c, max_* and *J_max_* in LJ but decreased in HJ (**Figure 5**). The increase in *V_c, max_* and *J_max_* was greater in LJ compared to SH and HJ, especially for intercellular CO_2_ concentrations above 400 ppm, consistent with the performance of photosynthetic rates, as can be observed from *A_n_*-*C_i_* curve (**Figure 4**). Similar results were also found in a study by Miao et al. (2021) who found that Yangdao6 (a more CO_2_-responsive indica cultivar) had a stronger *V_c, max_* and *J_max_* from heading to maturity under e[CO_2_] (+200) compared to other cultivars. Previous studies found that the response of *V_c, max_* and *J_max_* to climate change differed between species (Wullschleger, 1993). The various responses of *V_c, max_* and *J_max_* in C_3_ plants grown at e[CO_2_] were either positive such as *Liquidambar styraciflflua* (DeLucia and Thomas, 2000), or negative such as *Oryza sativa* L. and *Lolium perenne* L. (Ainsworth et al., 2003; Borjigidai et al., 2006). Interestingly, warming (1-2°C increase) and deficient soil nitrogen resulted in the decrease in *V_c, max_* and *J_max_* of rice and cotton species (Pettersson and McDonald, 1994; Cai et al., 2018). However, in the present study, the increased *V_c, max_* and *J_max_* in specific cultivars in response to e[CO_2_] and warming indicates its strong adaptability to climate change.

The *J_max_*: *V_c, max_* ratio is critical for understanding plant photosynthetic processes. (Miao et al., 2009). Compared to the *V_c, max_*, the response of *J_max_* to e[CO_2_] was more pronounced in this study. Some studies found that the enrichment of CO_2_ led to an increase of *J_max_*: *V_c, max_* ratio (Ainsworth and Long, 2005), but warming reduced this ratio (Kattge and Knorr, 2007; Lin et al., 2013). This is consistent with our findings that the *J_max_*: *V_c, max_* ratio was significantly increased under e[CO_2_] for three cultivars (**Figure 5**). Despite that [CO_2_] and temperature co-elevation only significantly increased the *J_max_*: *V_c, max_* ratio in SH, but had no remarkable effect on *J_max_*: *V_c, max_* ratio for HJ and LJ. It might explain the greater improvement in net photosynthetic rate for SH than HJ and LJ. Thus, the *J_max_*: *V_c, max_* ratio may be imperative to the instinct photosynthetic and yield performance under the changing environments.

### Leaf photosynthesis and water use efficiency under different treatments

The impact of e[CO_2_] on photosynthesis has been well documented in rice (Roy et al., 2012; Chen et al., 2014; Zhu et al., 2014; Cai et al., 2018). e[CO_2_] notably increased the net photosynthetic rate (*A_n_*) in three varieties, especially at the grain filling stage. These results were consistent with previous studies (Chen et al., 2014; Yuan et al., 2021; Wang et al., 2015a). While the average temperature at the test site did not exceed the suitable temperature for rice under the warming scenario in this study, but high temperatures and heat waves frequently occurred in July and August. This may increase photorespiration and respiration, which cause a reduction in the net photosynthesis rate (*A_n_*). Furthermore, Previous studies have shown that the anthesis stage of rice is more sensitive to environmental factors, such as temperature, etc. (Jagadish et al, 2007; Wassmann et al., 2009). Therefore, in the present study, a significant decrease in *A_n_* was found under warming at the anthesis stage, which may result in a decrease in rice yield formation or offset the positive effect of e[CO_2_] on rice yield.

In this study, the stomatal conductance was decreased with the continuous increase of intercellular CO_2_ concentration under [CO_2_] and temperature co-elevation (**Figure 2**). It is well recognized that increasing CO_2_ concentration led to a reduction in stomatal conductance (Bunce, 2004; Ainsworth and Rogers, 2007; Lenka et al., 2021a). However, previous studies indicated that the warming effect on stomatal conductance was either positive or negative (Mott and Peak, 2013; Lahr et al., 2015; Urban et al., 2017). This is likely attributed to the increase extent of the environmental temperature. Previous studies demonstrated that stomatal conductance was 4.3 times higher at 35°C than that at 15°C for the warm climate species such as soybean and tomato (Bunce, 2000a). However, several studies have also demonstrated that warming led to the reduction of stomatal conductance in rice and many other species (Lewis et al., 2002; Lahr et al., 2015; Göbel et al., 2019; Huang et al., 2021). These findings are in line with the present study. The differences in stomatal performance in response to warming may be related to the plant species or original habitats. Furthermore, the response of stomatal conductance to warming also varied with the phenological process, for instance, warming increased stomatal conductance for SH at the tillering stage except at anthesis and grain filling stages, indicating that stomatal conductance was not consistently characteristic in response to climate change throughout the growth phase (Wang et al., 2020b). Additionally, there was an offsetting effect on stomatal conductance under e[CO_2_] and warming at anthesis and grain filling stages (Del Pozo et al., 2005; Wang et al., 2020b).

The treatment of [CO_2_] and temperature co-elevation resulted in a higher water use efficiency (**Figures 3** and **9**). Numerous studies have shown that higher water use efficiency under e[CO_2_] (Bunce, 2000b; Allen et al., 2003; Ruiz-Vera et al., 2013; Li et al., 2019a). The water use efficiency can be mediated by both photosynthetic rate and transpiration rate, as previously observed in rice (Gauthami et al., 2014; Mishra et al., 2018). In the current study, under [CO_2_] and temperature co-elevation, the increase in water use efficiency is a result of a combination of increased photosynthetic rate and decreased transpiration rate, such as SH and LJ at the grain filling stage. Meanwhile, the water use efficiency of SH and LJ was more responsive to climate change than that of HJ, implying that improvement of water use efficiency under climate change could be improved by breeding new rice varieties in the future.

Stomatal conductance response to climate change may pose an essential role on the water use efficiency. A study in free air CO_2_ enrichment system (FACE) by Bernacchi et al. (2007) reported that e[CO_2_] decreased the evapotranspiration by 9-16% and as a result of the decrease in *G_s_*. Similarly, in a study on soybean in environment-controlled chambers, doubling [CO_2_] caused a 9% decline in evapotranspiration at a day/night temperature combination of 28/18°C, while there was the limited effect at 40/30°C and 44/34°C temperature treatments (Allen et al., 2003). In the present study, the transpiration rate of all cultivars was significantly decreased under e[CO_2_]. It may mainly be related to the significantly decreased stomatal conductance (Bernacchi et al., 2007; Kimball, 2016; Lenka et al., 2020). The reduction of stomatal conductance mainly occurred under e[CO_2_] rather than the warming condition (**Figures 2** and **7**), highlighting that enhanced water use efficiency under e[CO_2_] plus warming is the e[CO_2_]-induced reduction of stomatal conductance.

In the present study, the air -to- leaf temperature difference (*ΔT*) showed gradual decrease with the intercellular CO_2_ concentration increased under e[CO_2_], warming and e[CO_2_] plus warming (**Figure 10**). In general, a deceased stomatal conductance reduces leaf water loss and increases leaf temperature, resulting in a decrease in air-to-leaf temperature differences. (Allen et al., 2003; Kimball, 2016; Urban et al., 2017). The air -to- leaf temperature difference reduced under e[CO_2_] across three growth stages for all species. However, Lenka et al. (2020) reported that temperature increase above ambient 1.5°C along with e[CO_2_] was unlikely to lead to a difference in leaf temperature in soybean cultivar. In the present study, the air -to- leaf temperature difference in SH cultivar under e[CO_2_] plus warming is greater than that in HJ and LJ. The relatively stable response of the air -to- leaf temperature difference in HJ and LJ indicated a potential for physiological adaptation of rice cultivars to climate change.

### Warming alters biomass and yield under e[CO_2_]

Elevated temperature often reduces rice dry matter accumulation due to enhanced plant respiration, shortens growth duration and increases floral sterility (Ziska et al., 1996; Dong et al., 2011; Cai et al., 2016). However, warming has also been reported to benefit rice growth in regions with low ambient temperature (Keiichi et al., 2009; Adachi et al., 2014; Chen et al., 2017; Wang et al., 2020a). In the present study, warming promoted leaf photosynthesis, and panicle dry matter accumulation in SH and LJ at the stage of anthesis and grain filling. Additionally, there is substantial evidence that e[CO_2_] positively impacts rice dry matter accumulation which is consistent with the present study. (Sakai et al., 2001; Roy et al., 2012; Cai et al., 2018; Wang et al., 2020a). For example, the rice yield increased by 15% under e[CO_2_] (550 ppm) in a free-air [CO_2_] enrichment study (Wang et al., 2018). The results showed that while warming did not reduce or slightly reduced rice yield, the increase in yield under e[CO_2_] plus warming was less than that under e[CO_2_]. Nevertheless, considering the possible increase in rice biomass caused by the co-elevation of e[CO_2_] and temperature in this region, the rice varieties adapting to e[CO_2_] plus warming should receive more attention to improve rice production in the future.

## Conclusion

Warming by 2°C is likely to partially offset increases in photosynthetic rate attributed to e[CO_2_]. The [CO_2_] and temperature co-elevation may be favorable increasing photosynthetic ability of rice crop and improving water use efficiency. Although increased temperature in this study had a minor effect on grain yield, the effect may increase under future climate change. This study provided evidence that the rice has genotypic differences in photosynthetic potential under [CO_2_] and temperature co-elevation. Therefore, it is crucial to explore a broader range of phenotypes and cultivars in climate change adaption research, in order to advance the knowledge that climate change impacts rice crop resulting in a prospective increase of grain yield induced by [CO_2_] and temperature co-elevation in the cold-temperate climate regions.

## Data availability statement

The original contributions presented in the study are included in the article/supplementary material. Further inquiries can be directed to the corresponding authors.

## Author contributions

JJ and YL designed the experiments and managed the projects. YL, CZ, ZY, and JDL performed the experiments. CZ, KY, GW, XL, JLL and XZ performed the data analysis. JJ and CZ wrote the first draft of the manuscript, and YL and CZ edited and revised the manuscript. All authors contributed to the article and approved the submitted version.

## Funding

The project was funded by the Strategic Priority Research Program of the Chinese Academy of Sciences (XDA28100200, XDA28020201), the National Natural Science Foundation of China (32172123), Key Program of Natural Science Foundation of Heilongjiang Province of China (ZD2021D001), the International Partnership Program of Chinese Academy of Sciences (131323KYSB20210004), and Youth Innovation Promotion Association of Chinese Academy of Sciences (2019233), the International Partnership Program of Chinese Academy of Sciences (131323KYSB20210004), and Youth Innovation Promotion Association of Chinese Academy of Sciences (2019233).

## Acknowledgments

The authors are grateful to Drs. Ying Xu, Zhuxiu Liu and Haidong Gu for assisting with the data analysis, as well as Drs. Qingyun Bu and Xiufeng Li for providing the rice cultivar materials. We also appreciate the valuable work and comments of the reviewers and the editor.

## Conflict of interest

The authors declare that the research was conducted in the absence of any commercial or financial relationships that could be construed as a potential conflict of interest.

## Publisher’s note

All claims expressed in this article are solely those of the authors and do not necessarily represent those of their affiliated organizations, or those of the publisher, the editors and the reviewers. Any product that may be evaluated in this article, or claim that may be made by its manufacturer, is not guaranteed or endorsed by the publisher.
